# The impact of fatty acids as bioactive nutrients on the development of offspring

**DOI:** 10.3168/jdsc.2024-0654

**Published:** 2024-11-05

**Authors:** Ana C. Carranza-Martin, Donald L. Palmquist, Alejandro E. Relling

**Affiliations:** 1 IGEVET – Instituto de Genética Veterinaria “Ing. Fernando N. Dulout” (UNLP-CONICET La Plata), Facultad de Ciencias Veterinarias, Universidad Nacional de La Plata, CP 1900 La Plata, Buenos Aires, Argentina; 2 Department of Animal Sciences, The Ohio State University, Wooster, OH 44691

## Abstract

Fatty acids (FA) are normally considered a source of energy. However, some FA are essential nutrients with different biological functions, such as ligands for membrane and nuclear receptors; upon binding, they modify cell function and transcript expression. The bioactive effects of the FA depend on the FA type and family (i.e., n-6 vs. n-3). The FA effects on developmental programming have been studied in cattle and sheep, with some similarities in the outcomes between species. Feeding n-3 FA during late gestation improves offspring production performance (i.e., milk yield in dairy cows and growth in beef cattle and sheep) compared with the offspring of dams supplemented with mono- and unsaturated FA or with offspring of dams with no FA supplementation. Also, there is a sexual dimorphism in the outcomes of n-3 FA supplementation, where the increase in growth due to n-3 FA seems to be more evident in males, but it might decrease growth in females. There are multiple assumptions as to how this physiological process occurs. Based on published literature, the developmental effect does not appear to be due to changes in hypothalamic regulations of DMI and energy expenditure or liver and adipose tissue functions. The changes in offspring growth can be attributed to changes in gastrointestinal tract physiology, changes in immune response, or both, probably due to epigenetic changes in those tissues. Feeding n-3 FA in late gestation to the pregnant dam increases expression of amino acid transporters (mRNA and protein) in the offspring's duodenum, associated with changes in DNA methylation. Regarding immune function, the increase in offspring performance has been associated with decreased haptoglobin after weaning in calves or increases in lipid mediators, such as resolvin-D1 at birth. Supplementation with n-3 FA during late gestation affects offspring growth; changes in the offspring's gut and immune system biology can explain the sexual dysmorphism observed in changed body weight; however, we are unaware which of these basic mechanisms is responsible for the observed changes in biology.

## Highlights

• Fatty acid modulates activity of membrane and nuclear receptors.
• The fetal programming effect of n-3 fatty acids may be due to changes in energy partition.
• The fetal programming effect of n-3 fatty acids may change nutrient absorption.
• n-3 fatty acids modulate the immune system.
• n-3 fatty acids binding to receptors alters DNA methylation gene expression.

## Graphical Abstract

**Figure:**
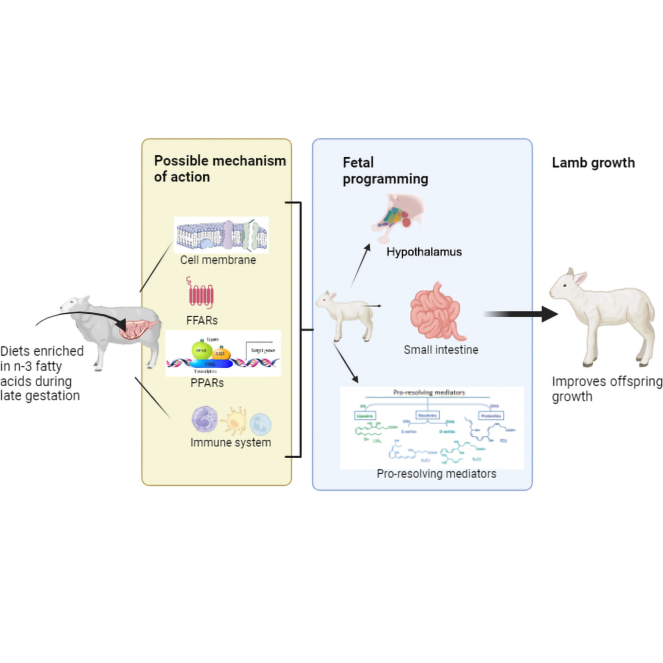
**Summary:** Feeding diets enriched with n-3 fatty acids during late gestation improves offspring growth in ruminants. The mechanisms of action are not fully elucidated. The increased growth has been associated with changes in transcription of genes associated with energy partitioning at the hypothalamus; however, there is some conflicting data on this mechanism of action. Fetuses and growing offspring from dams supplemented with n-3 fatty acids during late gestation had an increased number of amino acid transporters in the small intestine. Also, offspring born from dams supplemented with n-3 during late gestation showed an increase in resolvin D3, an anti-inflammatory lipid mediator, and a decreased concentration of haptoglobin during a stressful event. The increase in growth due to n-3 fatty acids may be due to the combination of the increased absorption of nutrients and regulating the immune system.

Fatty acids (**FA**) are organic molecules classified by their number of carbon atoms and number and position of double bonds between these carbon atoms (Maulucci et al., 2016). Fatty acids fulfill various functions, and they are important sources of energy. Also, FA are precursors for the biosynthesis of phospholipids, for the generation of numerous ligands for different types of receptors (Buczynski et al., 2009), and are the source of lipid mediators, particularly of molecules associated with inflammation (Norris and Dennis, 2014).

Traditionally, FA were added to ruminant diets to increase diet energy density, thus increasing energy intake to improve growth and production of animals (Santos et al., 2008). However, the effect PUFA on growth is not only due to the improvement of the animal's energy status but also to the bioactive functions of PUFA in different organs (Durkin et al., 2021). Furthermore, FA supplementation during gestation is associated with increased offspring growth, the expression of genes involved in lipid metabolism, cognitive and behavioral development, and energy metabolism in mammals (Palmquist, 2009).

The objective of this review is to describe the possible physiological mechanisms of how FA supplementation, particularly n-3 PUFA, during gestation can improve offspring production. To achieve this objective, we will first briefly describe the different mechanisms of action of FA. We will then discuss what is known about the effect of supplementation with FA during pregnancy on the growth of offspring. Last, we will describe the possible mechanisms of how PUFA cause the programming effect, and then conclude with some observations on what we consider interesting areas of future research on the topic.

To better understand the action of lipids in diverse tissues and cells, we have divided the effects of FA into 4 types of functions: (1) the activity of FA on membrane receptors, (2) the activity of FA on nuclear receptors, (3) the effects on cell membrane composition and fluidity, and (4) lipid mediators.

Fatty acids are ligands for membrane receptors. Four different free FA receptors (**FFAR**) have been described: FFAR1, FFAR2, FFAR3, and FFAR4. These receptors have different affinities for different FA (Kimura et al., 2020). The FFAR1 is stimulated by medium-length chain FA (6–12 C) and long-chain FA (13–21 C). This receptor is linked to a G protein subunit Gα_q/11_ and Gα_i/o_ signaling (Kimura et al., 2020). The FFAR4 is preferentially activated by long-chain FA and is coupled to a G protein Gα_q/11_ (Oh and Walenta, 2014). Free FA receptors 2 and 3 have a greater affinity for short-chain FA (2–6 C) and are coupled to Gα_i/o_ signaling (Grundmann et al., 2021). Due to the objective of this review, we will comment more on the functions of FFAR1 and 4. The activation of FFAR1 in the pancreas increases glucose-stimulated insulin secretion (Bharate et al., 2009) and glucagon secretion (Flodgren et al., 2007). The activation of FFAR1 and FFAR4 improves insulin sensitivity in pancreatic β cells and lymphocytes (Bharate et al. 2009; Vallée Marcotte et al., 2017). Additionally, FFAR4 activation increases adipogenesis (Hilgendorf et al., 2019) and lipogenesis (Ichimura et al., 2014. In the brain, activation of FFAR4/β-arrestin-2 leads to attenuation of the inflammatory pathway (Cintra et al., 2012). All FFAR are associated with intestinal motility and appetite, as they are found on L cells, which are responsible for the secretion of glucagon-like peptide 1 and peptide YY (De Silva and Bloom, 2012). The FFAR4 has been observed in the microvillus membrane of the human placenta (Lager et al., 2014). In reproductive tissue, the relative abundance of the FFAR4 mRNA transcript was observed in the cotyledon of sheep, being greater in ewes supplemented with eicosapentaenoic acid (**EPA**) and docosahexaenoic acid (**DHA**; Roque-Jiménez et al., 2020). The FFAR4 is known to play an important role in decidualization and maintenance of pregnancy in women (Huang et al., 2019).

The peroxisome proliferator-activated receptors (**PPAR**) are a group of nuclear protein receptors that promote ligand-dependent transcription of target genes that regulate energy production, lipid metabolism, and inflammation. The PPAR superfamily comprises 3 subtypes, PPARα, PPARγ, and PPARβ/δ, with different distributions among tissues. Each PPAR has a different effect on the regulation of energy balance and carbohydrate and lipid metabolism (Decara et al., 2020). The PPAR form a heterodimer with the retinoid-X receptor (Juge-Aubry et al., 1997) and, in target genes, binds to a peroxisome proliferator response element. In mammals, PPARα is expressed primarily in the liver and gastrointestinal tract. The PPARγ is found in white adipose tissue and immune cells. The PPARβ/δ is expressed ubiquitously (Auboeuf et al., 1997). In the endometrium, PPAR are involved in the regulation of prostaglandin, steroid, and cytokine synthesis (Bogacka et al., 2015). The PPARγ is highly expressed in the placenta and especially in the trophoblast, fulfilling its function in trophoblast differentiation. Embryos deficient in PPARγ fail to vascularize adequately (Zhu et al., 2000). Alterations in PPARβ/δ during early placental development decreased the strength of connections between the placenta and maternal decidua (Barak et al., 2002).

Cell membrane fluidity is defined as “the degree of molecular order and movement of membrane constituents, especially lipid components” (Hagve, 1988, p. 381). Membrane fluidity determines how easily lipids and proteins can diffuse laterally in the plane of the membrane (Ballweg et al., 2020). Several studies have shown that the amount of PUFA in phospholipids is the main factor regulating membrane fluidity (Hagve, 1988). Cell membranes contain more than a thousand different lipid species, but with a few exceptions, the clear function of each species remains largely unknown. Phospholipids constitute the main family, and although most cell membranes contain saturated and monounsaturated FA chains, certain organs such as the brain have a much greater percentage (∼30%) of phospholipids with PUFA (Ballweg et al., 2020). Neural membranes are highly enriched with DHA (PUFA n-3), constituting 30% to 40% of the phospholipids of the gray matter of the cerebral cortex (Innis, 2011). Changes in the balance between n-3 and n-6 PUFA alter the fluidity of the membrane. Additionally, dietary PUFA reduce cholesterol, which, when in excess, can cause neural membrane rigidity (Yehuda et al., 2002). Phospholipid species also serve as a reserve of PUFA, either for the short-term regeneration of species that have been degraded during membrane stimulation, or as a store for longer periods (Hagve, 1988).

Polyunsaturated FA are precursors of eicosanoids, which are key mediators and regulators of inflammation (Tilley et al., 2001). Some PUFA, such as arachidonic acid, EPA, and DHA, once released from membrane phospholipids, are enzymatically transformed by cyclooxygenases, lipoxygenases, and members of the cytochrome P450 family into numerous biologically active metabolites, such as prostaglandins, thromboxane, lipoxins, and eicosanoids (Norris and Dennis 2014). These molecules have different and even antagonistic effects depending on the type of cells and the stimulus that produced them, participating in the modulation of the intensity and duration of inflammatory responses (Tilley et al., 2001). Prostaglandin E_2_ is a potent inhibitor of the production of TNF-α and IL-1, classic proinflammatory cytokines released by monocytes and macrophages (Miles et al., 2002). Furthermore, prostaglandin-E_2_ inhibits lipoxygenase-5 (ALOX5) and induces lipoxygenase-15 (ALOX15), which promotes lipoxin formation (Levy et al., 2001) with anti-inflammatory effects (Vachier et al., 2002).

Polyunsaturated FA produce specialized pro-resolving mediators including resolvins, protectins, and maresins. The FA EPA produces E-series resolvins from a series of reactions involving cyclooxygenase-2 (COX-2) and ALOX5. These mediators appear to exert potent anti-inflammatory actions on neutrophils, macrophages, dendritic cells, and T cells (Serhan et al., 2000). In addition, DHA produces the D-series resolvins and protectins by a series of reactions involving ALOX enzymes. The D-series resolvin and protectins have anti-inflammatory action in neutrophils, macrophages, T cells, and microglia (Marcheselli et al., 2003).

A previous review (Roque-Jiménez et al., 2021) evaluated the effect of FA supplementation as a mechanism to program the development of the offspring. That review mainly described the effect of offspring growth due to FA supplementation during the different stages of developmental programming (pre-, during, and postgestational supplementation of FA). In this review, we will describe physiological changes that may be due to fetal programming. However, we will briefly mention some of the outcomes observed when FA are supplemented during gestation.

Supplementation with different sources of FA during different stages of gestation affects fetal growth differently. During early gestation (first third), lambs born from ewes fed greater amounts of saturated and monounsaturated FA have greater growth than lambs born from ewes supplemented with PUFA (Oviedo-Ojeda et al., 2021). However, the opposite effect has been observed in ruminants when supplementation occurred at the end of gestation (Marques et al., 2017; Carranza-Martin et al., 2018; Rosa-Velazquez et al., 2021; Rosa-Velazquez et al., 2022a). At the same time, no differences were observed when diets with different FA profiles were fed to ewes in mid-gestation (Rosa-Velazquez et al., 2022b). The reason for these differences in the different stages of gestation may be because of differences in tissue and organ development during different stages of gestation. It also appears that the time of supplementation of FA could affect the growth outcome of the offspring. When PUFA were supplemented in the last third of gestation during the winter, the offspring showed increased growth (Marques et al., 2017; Carranza-Martin et al., 2018; Brandão et al., 2020), but when pregnant dams were supplemented during summer, the opposite effect was observed (Shao et al., 2020). To add complexity to the model, there is a discrepancy in the effect of FA supplementation and outcome according to the sex of the offspring. These inconsistent results have been observed primarily in sheep, and the reason may be that beef cattle studies only work with steers (Marques et al., 2017; Brandão et al., 2020). In some studies, there is no sex by type of FA interaction observed on the growth of the offspring (Carranza-Martin et al., 2018); however, other results showed that male lambs have a greater growth rate than females when the mothers are supplemented with PUFA (Rosa-Velazquez et al., 2021).

Some of the mechanisms of action of the FA mentioned previously could be linked to developmental programming. It is believed that the intake of PUFA during pregnancy may contribute to the metabolic (or nutritional) programming of the fetus (Roque-Jiménez et al., 2021). However, the exact mechanisms of this discrepancy in the results of fetal programming are still unknown. Based on the cited literature, at least 3 different physiological mechanisms could be affected. These mechanisms are (1) changes in energy partitioning in the hypothalamus, (2) increased absorptive capacity of the offspring, and (3) changes in the magnitude of a response to inflammation.

During development, changes in hormonal signals and the intake of saturated and unsaturated FA alter the development of neuroregulatory signals that control feeding behavior (Innis, 2011). It is known that a high-fat maternal diet causes changes in hypothalamic peptides, such as proopiomelanocortin, agouti-related protein (AgRP), and neuropeptide Y (NPY) in the fetal brain (Page et al., 2009; Carranza-Martin et al., 2018). Supplementation with PUFA during the last third of pregnancy to the dam and PUFA supplementation to the growing lamb resulted in lambs with lower concentrations of mRNA of NPY, AgRP, ghrelin-receptor, NPY 1, and melanocortin receptor-4 mRNA (Carranza-Martin et al., 2018). However, data are contradictory on the effect of FA supplementation during late gestation, and data using RNA sequencing on the hypothalamus of FA-programmed lambs showed that prenatal PUFA supplementation had minimal impact on the hypothalamic gene expression (Rosa-Velazquez et al., 2024).

Despite limited published data, we consider that some of the differences in growth are associated with changes in the small intestine concentration of transporters for amino acids. We observed that supplementation of n-3 PUFA to ewes in late gestation increased the number of amino acid transporters in the small intestine (Rosa-Velazquez et al., 2020) in the fetus offspring. Those changes were associated with an increase in protein expression of amino acid transporters in adult lambs born from dams supplemented with different types of FA during gestation (Rosa-Velazquez et al., 2021).

Another mechanism that can explain the differences in growth of the offspring is the programming of the immune response. Dam supplementation with PUFA can modulate inflammatory mediators in the offspring (Marques et al., 2017; Rosa-Velazquez et al., 2022a). Supplementation with n-3 FA from wk 20 until delivery increased resolvin E and D precursors in the umbilical cord of women (See et al., 2017). Males born from ewes supplemented with n-3 PUFA from d 100 of gestation until parturition had greater a plasma resolvin D1 concentration than females (Rosa-Velazquez et al., 2021), whereas females born from ewes supplemented with a palmitic acid diet showed a greater plasma concentration of resolvin D1 compared with males. In addition, males born from ewes supplemented with n-3 PUFA had the highest concentration of resolvin D1. Finally, males born from mothers without FA supplementation showed the lowest concentration of resolvin D1 (Rosa-Velazquez et al., 2021). These differences were similar to the differences observed in the growth of that offspring. In another study in ewes fed from d 100 of gestation until birth with a control diet or diets supplemented with PUFA, methionine, or PUFA+methionine, it was observed in the liver of the 56 d old lambs that PUFA supplementation increased the mRNA concentration of resolvin-forming enzymes such as COX-2 and ALOX15 (Rosa-Velazquez et al., 2022a). In fetuses, dam PUFA supplementation increased the liver mRNA concentration of *ALOX5A*, which is related to resolvin-forming enzymes (Rosa-Velazquez et al., 2020). Studies conducted in cattle during late gestation have shown that steers born from PUFA-supplemented cows had a lower concentration of the acute phase protein haptoglobin (Marques et al., 2017) at weaning. Brandão et al. (2020) describe that in cows fed with a diet rich in PUFA 15 d before calving, the concentration of immunoglobulin G in the colostrum and the calf's plasma 24 h after birth were greater than in the control group fed saturated fat. In addition, the incidence of bovine respiratory disease decreased in calves born from PUFA-supplemented cows compared with the calves born from cows supplemented with saturated fat (Brandão et al., 2020).

In conclusion, the exact mechanisms of how n-3 PUFA supplementation during gestation improves offspring growth are not known. We assume that multiple mechanisms play a role in how maternal supplementation with n-3 PUFA affects the offspring's growth. These mechanisms are (1) changes in the absorption capability, which might be due to changes in DNA methylation of amino acids transporters genes; (2) modulation of the immune response; (3) changes on neuropeptides associated with energy partitioning; or (4) the interaction of any of these 3 mechanisms.
